# High expression of LEF1 correlates with poor prognosis in solid tumors, but not blood tumors: a meta-analysis

**DOI:** 10.1042/BSR20202520

**Published:** 2020-09-02

**Authors:** Xiaoqi Yang, Haoran Liu, Tao Ye, Zhangqun Ye

**Affiliations:** 1 Department of Urology, Tongji Hospital, Tongji Medical College, Huazhong University of Science and Technology, Wuhan 430030, P.R. China; 2 Hubei Institute of Urology, Wuhan 430030, P.R. China

**Keywords:** cancer, LEF1, meta-analysis, prognosis

## Abstract

**Background:** Previously published studies have indicated that lymphoid enhancer-binding factor 1 (LEF1) expression could be recognized as a valuable biomarker to evaluate clinical outcome for various types of malignant cancer, but the results remained controversial. Therefore, we conducted this meta-analysis to pool the published estimates and discuss the relationship of LEF1 expression with cancer prognosis. **Methods:** Five electronic databases Pubmed, Web of Science, Embase, CNKI, and Wanfang were systematically searched for eligible literatures. Hazard ratios (HRs) and 95% confidence intervals (CIs) from the included studies were combined to estimate the effect of LEF1 expression on cancer patients’ survival. **Results:** Eleven original studies met the criteria and were enrolled for analysis. The results indicated that compared with patients in low LEF1 expression group, patients in high LEF1 expression group tended to have shorter overall survival (HR = 1.74, 95% CI: 1.06–2.86, *P*=0.029), especially for patients with solid tumors (HR = 2.39, 95% CI: 1.86–3.08, *P*=0.000). **Conclusions:** Individual evidence about the prognostic value of LEF1 expression in human cancers was limited. Our meta-analysis supported the suggestion that elevated LEF1 expression could function as a promising biomarker to predict the clinical outcomes for malignant cancers, especially solid tumors. More high-quality clinical studies are warranted to highlight the prognostic value of LEF1 expression in human cancers.

## Introduction

As a major cause of mortality worldwide, malignant cancer has become a huge health problem for human beings [1]. The global burden of human tumors is projected to increase largely because of population aging and the absence of efficient early detection methods [2]. Despite the rapid progress in the management and treatment of cancers, the survival rate of cancer patients remains low [3]. Therefore, identification of novel reliable biomarkers is urgently needed to help reduce cancer-related deaths.

Lymphoid enhancer-binding factor 1 (LEF1) is a member of high mobility group family, mainly involved in the transcriptional regulation of gene expression by altering the structure of DNA helix [4,5]. Wnt/β-catenin pathway plays a critical role in the tumorigenic properties of many cancers. As a downstream regulator of Wnt/β-catenin pathway, LEF1 was reported to contribute to stem cell maintenance, epithelial–mesenchymal transition (EMT), and tumor invasion [6–9]. Recent years, many studies have reported aberrant expression of LEF1 in various types of cancer, including solid tumors (esophageal squamous cell carcinoma (ESCC), prostate cancer, and hepatocellular carcinoma (HCC)) [10–12] and blood tumors (acute myeloid leukemia (AML), acute promyelocytic leukemia (APL), and acute lymphoblastic leukemia (ALL)) [13–15]. In addition, the prognostic significance of LEF1 expression in human cancers has attracted a lot of interest; however, the previously published studies displayed conflicting results, especially the obvious differences between solid and blood tumors. Some studies reported LEF1 overexpression correlated with unfavorable prognosis in several solid tumor types, including ESCC, nasopharyngeal carcinoma (NPC), and colorectal cancer (CRC) [16–18]; while other studies found a positive relationship of LEF1 expression with favorable prognosis in blood tumors, such as AML and ALL [15,19]. However, contradictory results were also reported. Jia et al. affirmed that the mRNA level of LEF1 in children ALL patients was significantly higher compared with those in normal controls, and high LEF1 level was related to favorable complete remission rate and better survival [15]; but in another study, Kuhnl et al. found LEF1 overexpression was associated with worse outcomes in adult ALL patients [20]. Due to relatively small number of cancer patients in a single study, we performed this meta-analysis based on all current evidence to reach a more reliable conclusion about the prognostic value of LEF1 overexpression in human cancers.

## Materials and methods

### Literature search

Five electronic databases Pubmed, Web of Science, Embase, CNKI, and Wanfang were systematically searched for studies in human exploring the relationship between LEF1 expression and prognosis of various malignant cancers. The following terms were used as search keywords with varied combinations: (‘LEF1’ OR ‘Lymphoid enhancer-binding factor 1’ OR ‘lymphoid enhancer factor 1’) AND (‘cancer’ OR ‘tumor’ OR ‘carcinoma’) AND (‘prognosis’ OR ‘survival’) covering all articles published in English up to August 2019. The reference lists were carefully reviewed for additional eligible studies.

### Inclusion and exclusion criteria

Eligible articles meeting the following criteria were enrolled in this meta-analysis: (i) studies investigated human malignant cancers and enrolled more than 50 participants; (ii) the relationship of LEF1 expression with cancer prognosis was reported; (iii) studies directly reported hazard ratios (HRs) with their 95% confidence intervals (CIs) of overall survival (OS) and recurrence-free survival (RFS). Abstracts, case reports, reviews, animal studies, and studies without sufficient data were excluded.

### Data extraction and quality assessment

The quality of articles satisfying all inclusion criteria was first assessed by two independent reviewers following the Newcastle–Ottawa scale (NOS) scoring system and score ≥ 6 was considered high quality [21]. The following data from all eligible studies were collected: name of first author, year of publication, country of origin, cancer type, study design, number of cancer patients, age of patients, detection method, cut-off value, follow-up time, analysis method, and HR value with their 95% CI of OS and RFS from multivariate or univariate analysis. Disagreements between authors were adjudicated by discussing with a third reviewer.

### Statistical analysis

HRs with 95% CIs were pooled to evaluate the impact of LEF1 expression on prognosis of cancer patients (OS and RFS, respectively). Statistical heterogeneity among the included studies was measured using *I^2^* statistics and Chi-square based Q test. If the *P*-value of Q test < 0.10 or *I^2^* > 50%, a random-effects model was used; if the *P*-value of Q test ≥ 0.10 or *I^2^* ≤ 50%, a fixed-effects model was applied. Besides, Galbraith plot was used to explore the origin of the significant heterogeneity. Sensitivity analysis was conducted to check the stability of the final results and Begg’s test to determine the risk of publication bias among the included studies. All analyses were conducted with Stata 12.0 software (Stata Corp., College Station, U.S.A.). *P*-value less than 0.05 was considered to be statistically significant.

## Results

### Literature search and study characteristics

**Figure 1:**
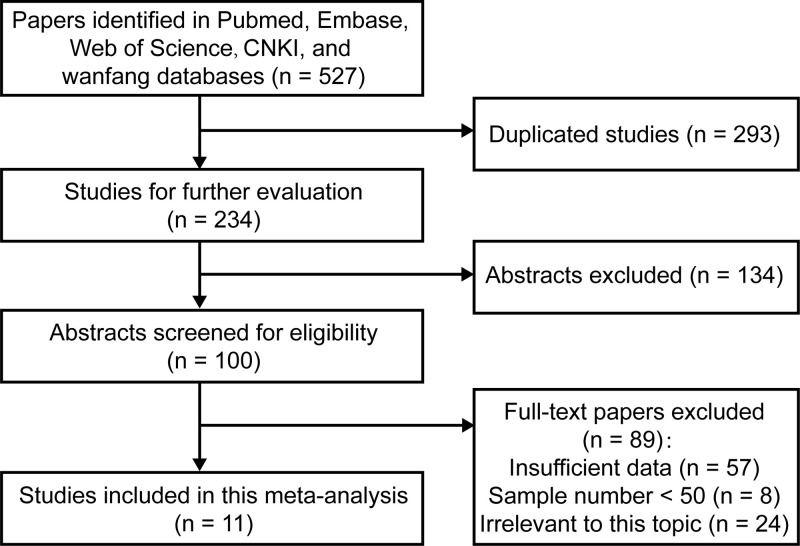
Flow diagram showed the selection process of included studies

The detailed process of study selection is shown in Figure 1. A total of 527 potentially relevant publications were initially obtained based on the search strategies, and then 234 studies remained after duplicates excluded. Through carefully screening the titles and abstracts, 134 articles were discarded for not reporting the association of LEF1 expression with cancer prognosis. For the remaining 100 records, after systematically reviewing the full-texts, another 89 studies were excluded (57 for insufficient data, 8 for sample number < 50, and 24 for irrelevant to this topic), and eventually, 11 eligible case–control studies with 1966 cancer patients were included in this current meta-analysis [13–20,22–24]. These studies were published from 2010 to 2019, and six studies were conducted in China, one in U.S.A., three in Germany, and one in Italy. Seven different types of malignant cancer were investigated, including AML (two studies), CRC (three studies), NPC, oral squamous cell carcinoma (OSCC), APL, ALL (two studies), and ESCC. The number of patients in individual study ranged from 78 to 391. All blood tumor studies measured LEF1 expression in bone marrow samples, while solid tumor studies in tissue samples. The expression level of LEF1 was detected by quantitative real-time polymerase chain reaction (qRT-PCR) in five studies, tissue microarray (TMA) in two studies, and immunocytochemistry (IHC) in four studies. For the disease outcomes, ten studies reported OS in multivariate analysis, three studies reported OS in univariate analysis, and four studies reported RFS in multivariate analysis. The main characteristics and data related to outcomes were detailed in Tables 1 and 2. Using the NOS scoring system, all eligible studies were regarded as high quality (Table 3).

**Table 1:** Main characteristics of the included studies

First author	Year	Country	Cancer type	Study design	Number of patients	Age, years	Samples	Method	Cut-off value	Outcome
Fu	2014	China	AML	Case–control	101	47 (13–85)	Bone marrow	qRT-PCR	Median	OS, RFS
Lin	2011	USA	CRC	Case–control	154	62 (27– 92)	Tissue	TMA	Score ≥ 2	OS
Zhan	2019	China	NPC	Case–control	391	NR	Tissue	IHC	Score ≥ 2	OS
Su	2013	China	OSCC	Case–control	135	NR	Tissue	IHC	>5% tumor cells	OS
Metzeler	2016	Germany	AML	Case–control	210	59 (17– 83)	Bone marrow	qRT-PCR	ΔΔ*C*_T_ value of −2.50	OS, RFS
Wang	2013	China	CRC	Case–control	184	54 (30– 78)	Tissue	IHC	Score ≥ 5	OS
Kriegl	2010	Germany	CRC	Case–control	214	NR	Tissue	TMA	Score ≥ 2	OS
Albano	2013	Italy	APL	Case–control	78	45 (16– 88)	Bone marrow	qRT-PCR	Median	OS
Kuhnl	2011	Germany	ALL	Case–control	282	NR	Bone marrow	qRT-PCR	>75% expression level	RFS
Jia	2015	China	ALL	Case–control	122	6 (1–14)	Bone marrow	qRT-PCR	Median	OS, RFS
Zhao	2019	China	ESCC	Case–control	95	NR	Tissue	IHC	Score ≥ 2	OS

Abbreviation: NR, not reported.

**Table 2:** Synthesis of data extracted from the included studies related to outcomes pooled in the meta-analysis

First author	Year	Follow-up, months	Analysis method	OS		RFS	
				HR	95% CI	HR	95% CI
Fu	2014	7.3 (0.3–47)	Multivariate	1.54	0.20–11.78	2.03	0.27–15.22
Lin	2011	36 (7.2–183.6)	Univariate	1.66	1.04–2.63	NR	NR
			Multivariate	1.78	1.09–2.89	NR	NR
Zhan	2019	NR	Multivariate	3.00	1.86–4.84	NR	NR
Su	2013	≤ 144	Univariate	1.96	1.02–3.75	NR	NR
			Multivariate	2.08	1.13–3.84	NR	NR
Metzeler	2016	47^1^	Multivariate	0.60	0.40–0.90	0.50	0.30–0.83
Wang	2013	≤60	Univariate	2.31	1.15–4.64	NR	NR
			Multivariate	2.67	1.31–4.85	NR	NR
Kriegl	2010	≤150	Multivariate	2.66	1.11–6.34	NR	NR
Albano	2013	68.4^1^	Multivariate	3.30	1.00–10.50	NR	NR
Kuhnl	2011	42.6^1^	Multivariate	NR	NR	2.40	1.10–5.10
Jia	2015	67.2^1^	Multivariate	0.44	0.20–1.01	0.68	0.35–1.31
Zhao	2019	27^1^	Multivariate	3.06	1.13–8.26	NR	NR

Abbreviation: NR, not reported.

1 Median follow-up.

**Table 3:** Quality assessment of eligible studies with NOS

First author	Year	NOS	Selection	Comparability	Outcome
Fu	2014	6	★★	★★	★★
Lin	2011	7	★★	★★	★★★
Zhan	2019	7	★★★	★★	★★
Su	2013	8	★★★	★★	★★★
Metzeler	2016	8	★★★	★★	★★★
Wang	2013	8	★★★	★★	★★★
Kriegl	2010	7	★★★	★★	★★
Albano	2013	7	★★	★★	★★★
Kuhnl	2011	7	★★★	★★	★★
Jia	2015	8	★★★	★★	★★★
Zhao	2019	7	★★	★★	★★★

### LEF1 expression and OS

**Figure 2:**
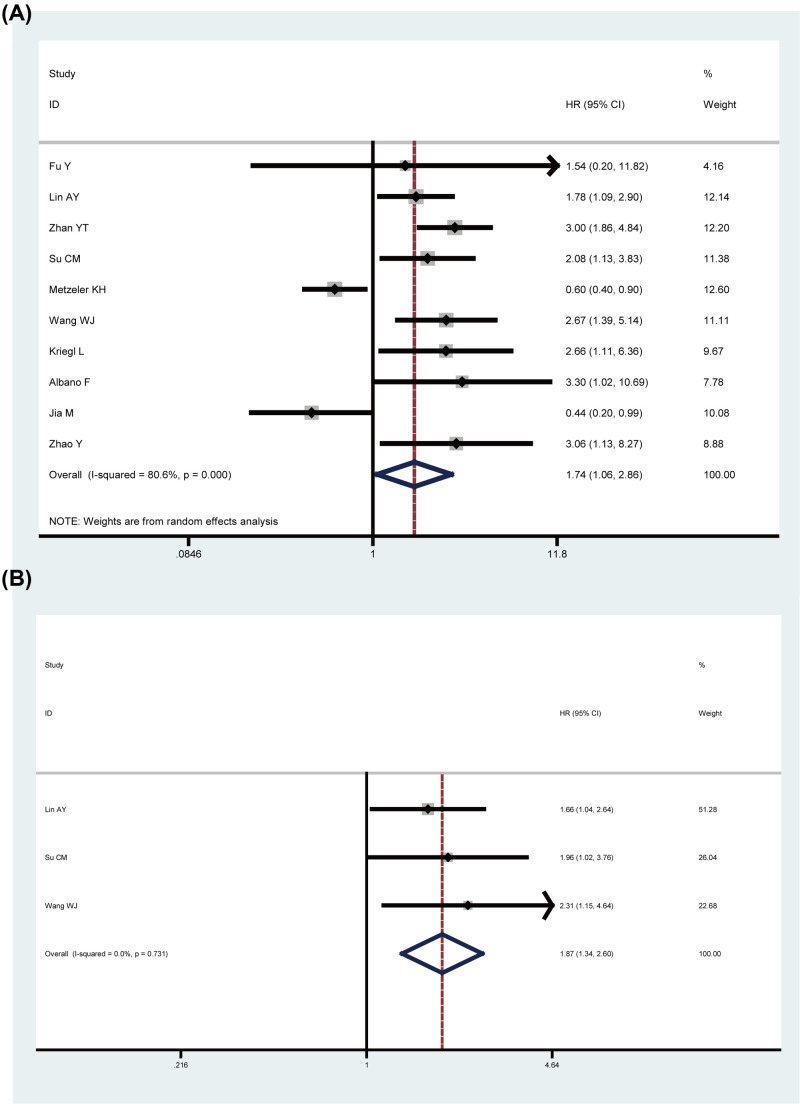
Forest plots for the relationship between LEF1 expression and OS (**A**) Meta-analysis of OS in multivariate analysis. (**B**) Meta-analysis of OS in univariate analysis.

Ten articles reported the impact of LEF1 expression on OS using HRs with 95% CIs in multivariate analysis with a total of 1684 patients diagnosed with human cancers. Due to the obvious heterogeneity among these studies (*I^2^* = 80.6%, *P*=0.000), a random-effects model was used and a significant relationship was observed between elevated LEF1 expression and poor OS of cancer patients (HR = 1.74, 95% CI: 1.06–2.86, *P*=0.029) (Figure 2A). In addition, three studies with 473 cancer patients reported HRs with 95% CIs in univariate analysis. A fixed-effects model was applied (*I^2^* = 0, *P*=0.731) and the pooled HRs were 1.87 (95% CI: 1.34–2.60, *P*=0.000) (Figure 2B). These results indicated that cancer patients with high LEF1 expression tended to have a worse OS.

**Figure 3:**
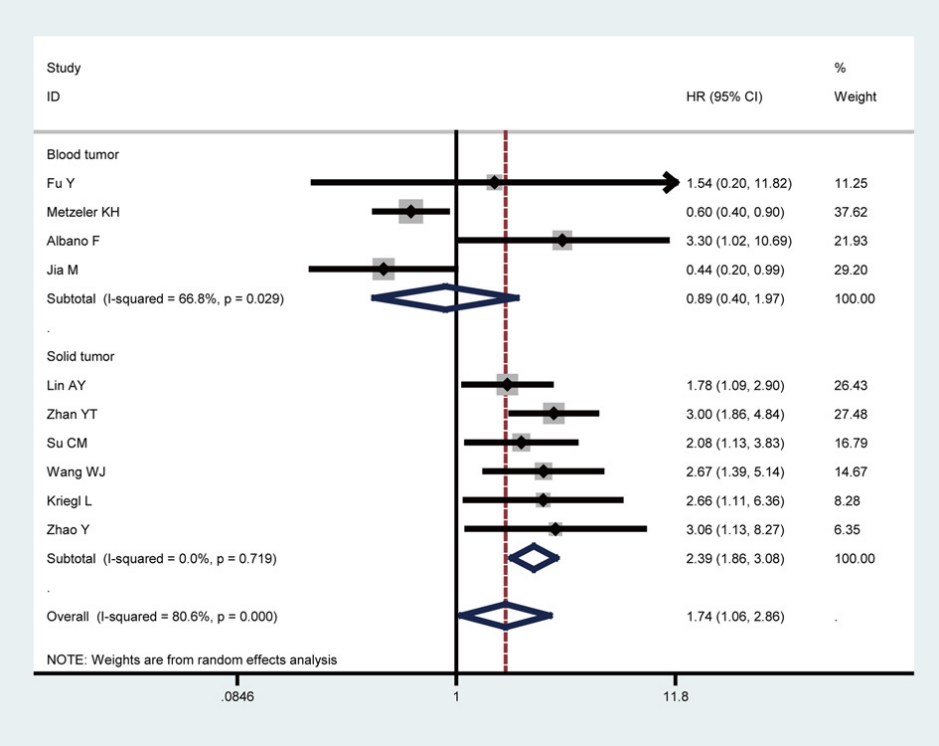
Subgroup analyses of OS by blood or solid tumors

**Figure 4:**
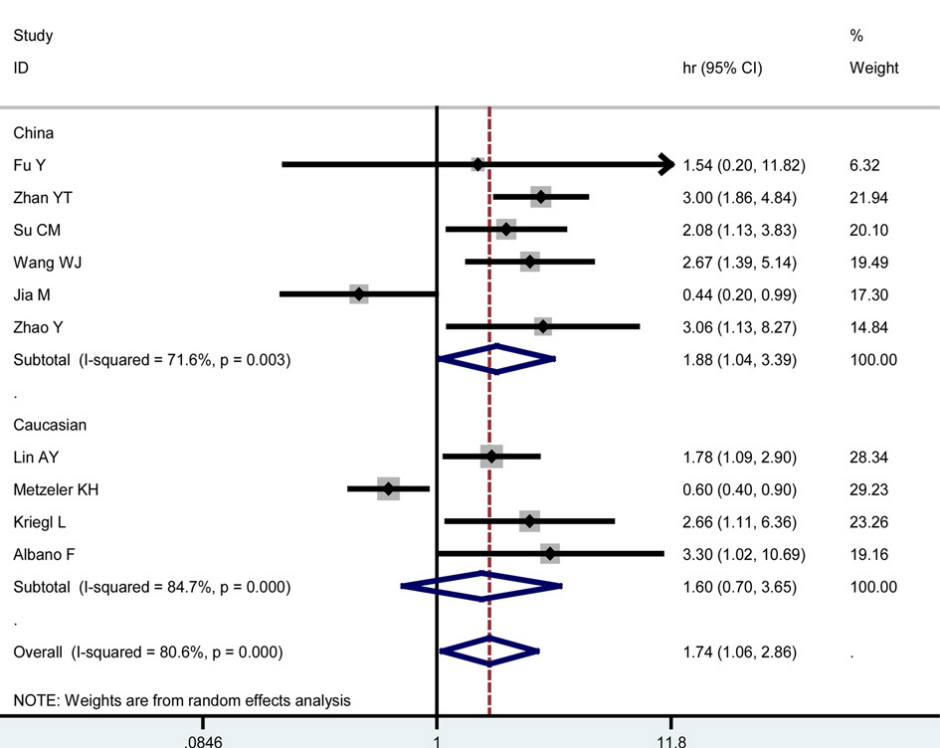
Subgroup analyses of OS by ethnicity

To explore the difference about the prognostic value of LEF1 overexpression for OS between solid and blood tumors, subgroup analysis was conducted. It was found that increased LEF1 expression was positively correlated with shorter OS in solid tumors (HR = 2.39, 95% CI: 1.86–3.08, *P*=0.000), but not in blood tumors (HR = 0.89, 95% CI: 0.40–1.97, *P*=0.076) (Figure 3). Therefore, LEF1 expression might serve as a predictor for unfavorable OS among patients with solid tumors. With respect to a certain cancer type, a significant correlation was identified between LEF1 expression and OS in patients with CRC (HR = 2.15, 95% CI: 1.50–3.07, *P*=0.000) (Supplementary Figure S1). Furthermore, a significant association was observed between overexpressed LEF1 and poor OS in Chinese cancer patients (HR = 1.88, 95% CI: 1.04–3.39, *P*=0.036), but not in Caucasian cancer patients (*P*=0.263) (Figure 4). As for the obvious heterogeneity among these studies in the meta-analysis of OS in multivariate analysis, the results of Galbraith plot (Supplementary Figure S2) and subgroup analyses suggested that Zhan study, Metzeler study, and Jia study might be the origin of heterogeneity.

### LEF1 expression and RFS

**Figure 5:**
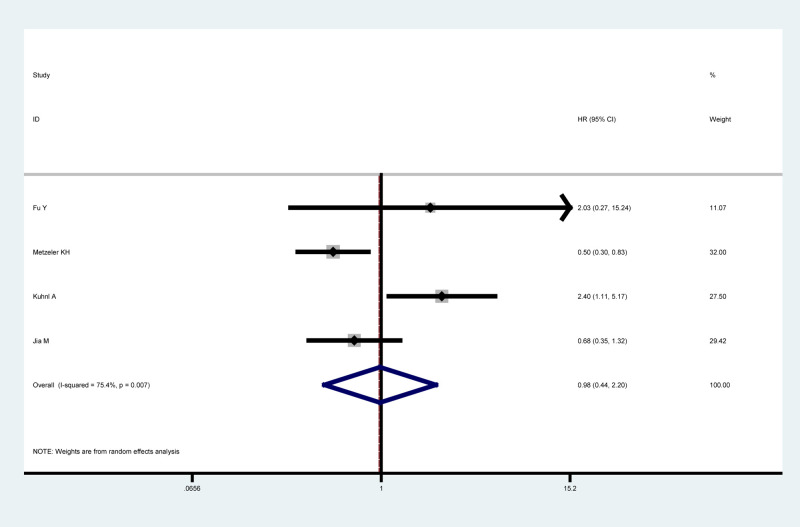
Forest plot for the relationship between LEF1 expression and RFS

The relationship between LEF1 expression and RFS was reported in four articles with a total of 715 patients, all these studies were conducted in patients with blood tumors and reported HRs with 95% CIs of RFS in multivariate analysis. The heterogeneity test showed significant heterogeneity (*I^2^* = 75.4%, *P*=0.007) and a random-effects model was applied. However, the results failed to identify a significant relationship between LEF1 expression and RFS (HR = 0.98, 95% CI: 0.44–2.20, *P*=0.969) (Figure 5).

### Sensitivity analysis and publication bias

**Figure 6:**
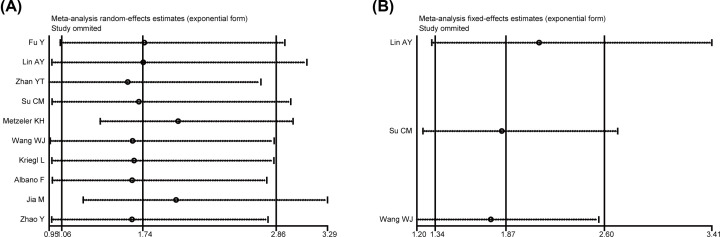
Sensitivity analyses for the meta-analysis of OS (**A**) Sensitivity analysis for the meta-analysis of OS in multivariate analysis. (**B**) Sensitivity analysis for the meta-analysis of OS in univariate analysis.

**Figure 7:**
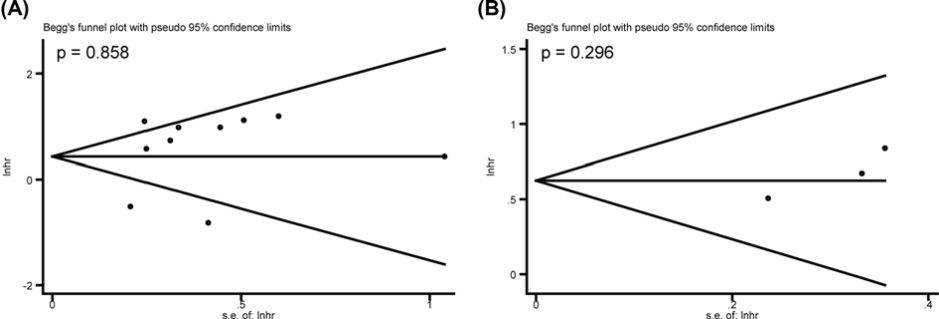
Begg’s tests for the meta-analysis of OS (**A**) Begg’s tests for the meta-analysis of OS in multivariate analysis. (**B**) Begg’s tests for the meta-analysis of OS in univariate analysis.

As illustrated in Figure 6A,B, sensitivity analyses were performed for the meta-analysis results of OS in both multivariate and univariate analyses, the results confirmed the robustness about the predictive value of high LEF1 expression on unfavorable OS. Begg’s tests were used to detect the publication bias, and no significant publication bias was observed in the meta-analysis of OS in both multivariate (*P*=0.858, Figure 7A) and univariate (*P*=0.296, Figure 7B) analyses.

## Discussion

LEF1 is a critical transcription factor primarily regulating the Wnt/β-catenin signaling pathway and involved in the oncogenesis and progression of numerous malignancies [25]. Aberrant LEF1 expression has been discovered in various types of human cancer, and correlated with poor survival. In CRC, LEF1 overexpression was positively detected in 44% of cancer patients by TMA analysis and significantly correlated with shorter OS and subsequent occurrence of liver metastasis [22]. In patients with NPC, 43.5% of the cases (170/391) showed positive expression of LEF1; additionally, LEF1 expression was associated with poor OS and several clinicopathological features (including lymph node metastasis and advanced clinical stage) [17]. Su et al. [23] discovered elevated LEF1 expression in 24% of the OSCC patients (33/135), and multivariate analysis result showed that LEF1 expression might serve as a predictor for unfavorable prognosis. These above-mentioned studies all explored the prognostic value of LEF1 expression in solid tumors, and mainly revealed a positive relationship of increased LEF1 expression with poor prognosis.

In addition, plenty of other clinical researches discussed the impact of LEF1 expression on blood tumors. Fu et al. [13] detected higher LEF1 mRNA level in previously untreated AML patients compared with healthy controls, and patients with elevated LEF1 expression probably had better treatment response for the initial induction therapy. Metzeler et al. [19] reported that AML patients with high LEF1 status had a trend toward prolonged RFS (*P*=0.007) and OS (*P=*0.01). Similarly in ALL, children patients tended to have higher LEF1 expression than the normal controls, and the high LEF1 expression group had an obviously favorable complete remission rate and longer OS, but the multivariate analysis failed to identify LEF1 expression as an independent prognostic predictor of better OS for not reaching statistical significance (*P*=0.052) [15]. In chronic lymphocytic leukemia (CLL), LEF1 expression was usually undetected in normal mature B cells, but observed in B cells of CLL patients; Tanden et al. [26] reported that all their 92 research patients showed strong nuclear LEF1 expression. It was affirmed that CLL patients with high LEF1 expression exhibited unfavorable prognosis than those with low LEF1 expression [27]. Accordingly, these studies showed LEF1’s prognostic value in human blood tumors was controversial. Considering the fact that the LEF1 level of bone marrow samples in blood tumors was influenced by the percentage of the malignant hematopoietic cells and the stage of the leukemia, it might result in relatively decreased LEF1 level in bone marrow samples of leukemia. This might explain the different results about the prognostic value of LEF1 in leukemia.

To our knowledge, this was the first meta-analysis which focused on the effect of elevated LEF1 expression on the prognosis of cancer patients. Eleven eligible studies comprising 1966 cancer patients were selected and analyzed in this work. The pooled results of multivariate analyses suggested that higher LEF1 expression was significantly related to poorer OS; likewise, similar results were achieved in univariate analyses. These results summarized that increased LEF1 expression could be used to predict unfavorable prognosis for cancer patients. To explore more detailed conclusions, these eligible studies were stratified for further analyses. Four studies were divided into blood tumor subgroup (HR = 0.89, 95% CI: 0.40–1.97) and six studies into solid tumor subgroup (HR = 2.39, 95% CI: 1.86–3.08). Accordingly, the prognostic value of LEF1 expression might be more significant in solid tumors, while it was still hard to draw accurate conclusion for these conflicting results in the four enrolled studies of blood tumors. Moreover, it was worth mentioning that we unexpectedly learned that Chinese cancer patients with high LEF1 expression had worse OS than those with low expression. Considering the significant heterogeneity observed, Galbraith plot was performed, combining with the results of subgroup analyses, three studies (Zhan study, Metzeler study, and Jia study) were considered to be the potential source of heterogeneity. The heterogeneity might be mainly due to the simultaneous inclusion of both solid and hematologic tumors, and the inconsistent characteristics of enrolled participants.

Besides, several limitations are also needed to be noted. First, the number of eligible studies was relatively small, which might influence the pooled results, especially larger scale studies are needed to further discuss the correlation between LEF1 expression and the prognosis of blood tumors. Second, studies with positive results were more likely to be published, and this might lead to publication bias causing overestimation of the final results. Third, different techniques were used to determine the LEF1 expression in the included studies and the cutoff value for positive LEF1 expression was not always consistent. Finally, although no obvious difference was identified by sensitivity analyses and Begg’s tests, publication bias still could not be utterly cleared.

In conclusion, it was shown by our meta-analysis that elevated LEF1 expression was correlated with poorer OS in patients with various types of cancer, especially solid tumors. LEF1 expression was suggested to serve as a promising biomarker for predicting prognosis in cancer patients. In the future, more standardized and larger-scale studies are eagerly warranted to reach a more persuasive conclusion.

## Supplementary Material

Supplementary Figures S1-S2

## Abbreviations

ALL: acute lymphoblastic leukemia
AML: acute myeloid leukemia
APL: acute promyelocytic leukemia
CI: confidence interval
CLL: chronic lymphocytic leukemia
CRC: colorectal cancer
ESCC: esophageal squamous cell carcinoma
HR: hazard ratio
IHC: immunocytochemistry
LEF1: lymphoid enhancer-binding factor 1
NOS: Newcastle–Ottawa scale
NPC: nasopharyngeal carcinoma
OS: overall survival
OSCC: oral squamous cell carcinoma
RFS: recurrence-free survival
TMA: tissue microarray
