# Supplementary material for: High expression of LEF1 correlates with poor prognosis in solid tumors, but not blood tumors: a meta-analysis
Source: Biosci Rep. 2020 Sep 2;40(9):BSR20202520. doi: 10.1042/BSR20202520 (PMC7468095; doi:10.1042/BSR20202520)
Supplement: Supplementary Figures S1-S2 [file BSR-2020-2520_supp.pdf]

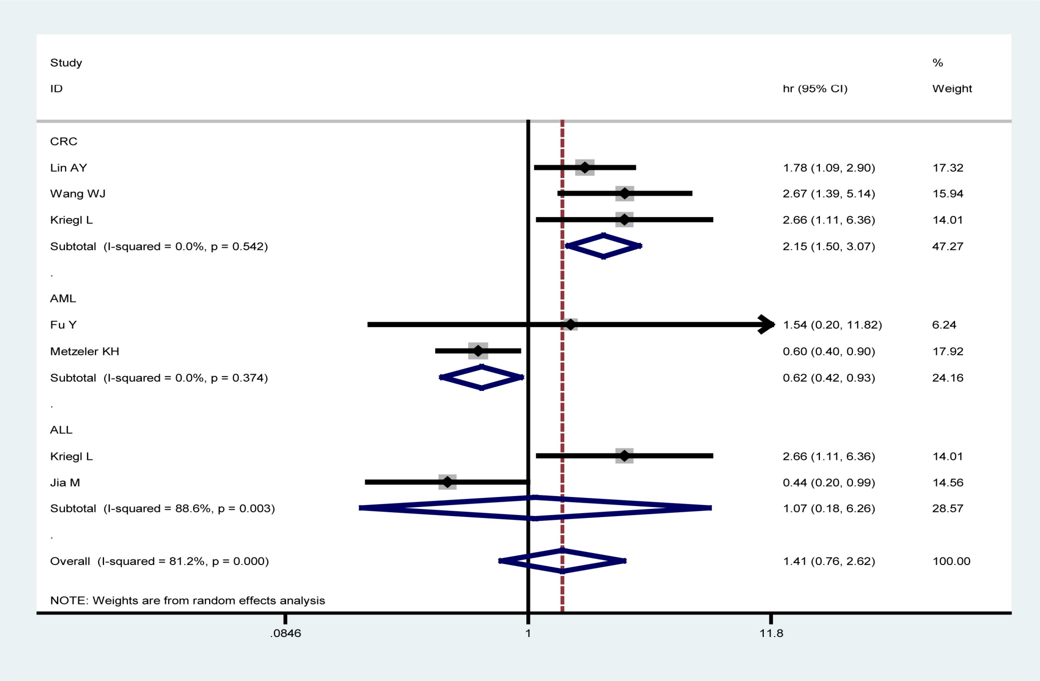

Figure S1. Subgroup analyses of OS by certain cancer type.

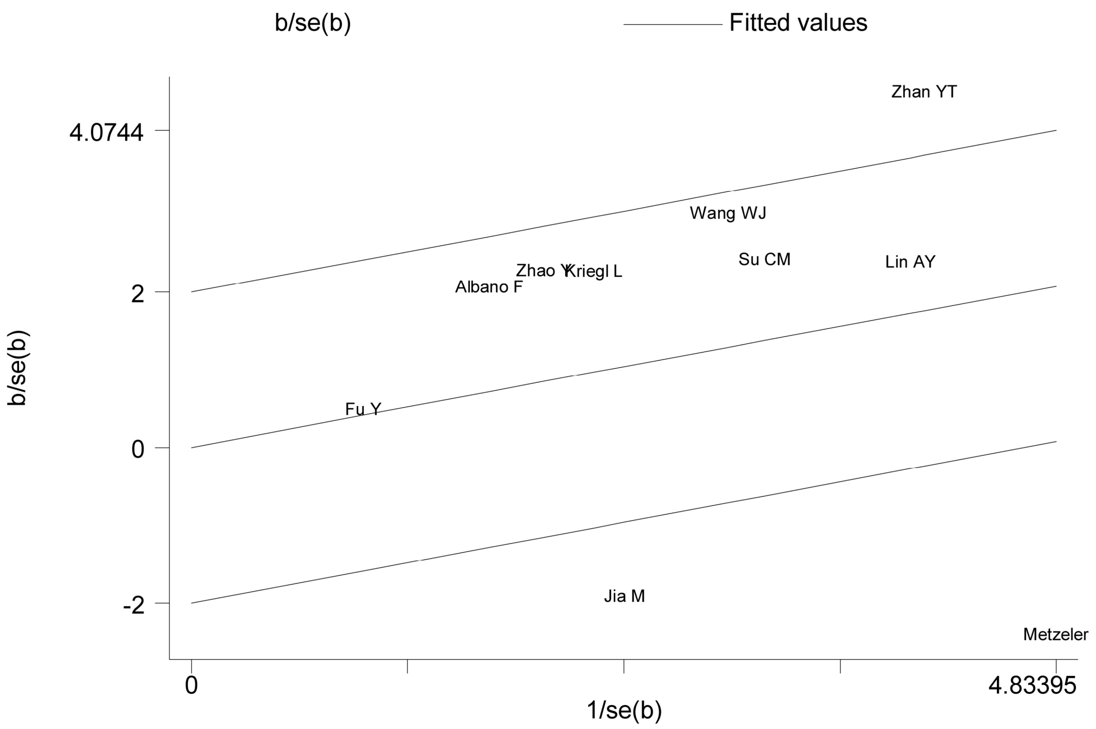

Figure S2. Galbraith plot for the relationship between LEF1 and OS.
